# Current Concepts of Hyperinflammation in Chronic Granulomatous Disease

**DOI:** 10.1155/2012/252460

**Published:** 2011-07-25

**Authors:** Nikolaus Rieber, Andreas Hector, Taco Kuijpers, Dirk Roos, Dominik Hartl

**Affiliations:** ^1^Children's Hospital, University of Tübingen, 72076 Tübingen, Germany; ^2^DFG Emmy-Noether Group, Research Center, Children's Hospital, Ludwig-Maximilians-University, 80337 Munich, Germany; ^3^Sanquin Research Institute, Landsteiner Laboratory, 1006 AD Amsterdam, The Netherlands; ^4^Emma Children's Hospital, Academic Medical Centre, University of Amsterdam, 1105 AZ Amsterdam, The Netherlands

## Abstract

Chronic granulomatous disease (CGD) is the most common inherited disorder of phagocytic functions, caused by genetic defects in the leukocyte nicotinamide dinucleotide phosphate (NADPH) oxidase. Consequently, CGD phagocytes are impaired in destroying phagocytosed microorganisms, rendering the patients susceptible to bacterial and fungal infections. Besides this immunodeficiency, CGD patients suffer from various autoinflammatory symptoms, such as granuloma formation in the skin or urinary tract and Crohn-like colitis. Owing to improved antimicrobial treatment strategies, the majority of CGD patients reaches adulthood, yet the autoinflammatory manifestations become more prominent by lack of causative treatment options. The underlying pathomechanisms driving hyperinflammatory reactions in CGD are poorly understood, but recent studies implicate reduced neutrophil apoptosis and efferocytosis, dysbalanced innate immune receptors, altered T-cell surface redox levels, induction of Th17 cells, the enzyme indolamine-2,3-dioxygenase (IDO), impaired Nrf2 activity, and inflammasome activation. Here we discuss immunological mechanisms of hyperinflammation and their potential therapeutic implications in CGD.

## 1. Chronic Granulomatous Disease

Chronic granulomatous disease (CGD) is the most common inherited disorder of phagocytic functions, caused by genetic defects in the leukocyte nicotinamide dinucleotide phosphate (NADPH) oxidase. Phagocytes of CGD patients are unable to kill ingested microorganisms through reactive oxygen species (ROS), resulting in an augmented susceptibility of the patients to bacterial and fungal infections [1, 2]. Most CGD patients are known to suffer from recurrent infections from the first year of life. To prevent severe life-threatening infections, these patients require prophylactic antibiotics and antimycotics [2, 3]. Bone marrow transplantation is considered curative, and gene therapy approaches have also been implemented to overcome severe infections in CGD [4–8]. In addition to their impaired antibacterial and antifungal innate host defense, CGD patients frequently present with autoimmune phenomena, such as granuloma formation, Crohn-like disease, pulmonary fibrosis, or others, suggesting a dysbalanced uncontrolled inflammatory response in CGD [3]. Today, most of the CGD patients reach adulthood due to improved treatment options for the bacterial and fungal infections, yet the autoinflammatory manifestations become more prominent. The severe recurrent infections can be explained by the lack of ROS required for the killing of pathogens, whereas the underlying reasons for the hyperinflammatory reactions in CGD remain poorly understood, and causative therapy for CGD hyperinflammation is lacking. 

## 2. Inflammation in Chronic Granulomatous Disease

Several lines of evidence support the concept that CGD features hyperinflammation [9]. Notably, microarray analysis in neutrophils from CGD patients has revealed upregulation of several proinflammatory genes [10]. Upon stimulation with TLR2 or TLR4 ligands, leukocytes from CGD patients yield an increased production of proinflammatory cytokines, which is, surprisingly, independent of NADPH oxidase activity [11].

### 2.1. Neutrophil Apoptosis and Efferocytosis

**Figure 1:**
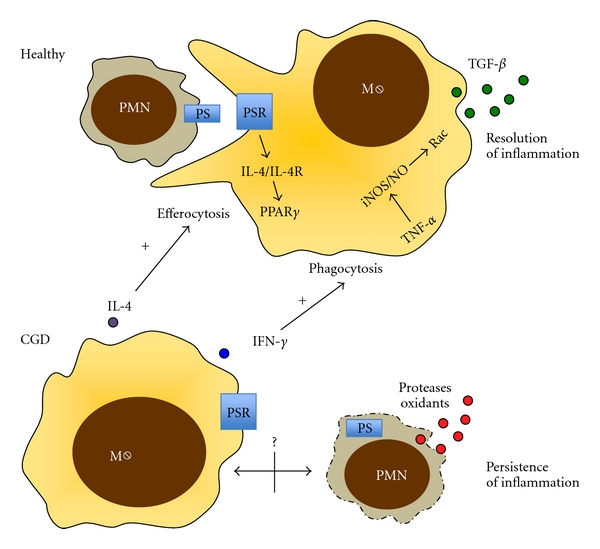
Impaired apoptosis/efferocytosis underlying hyperinflammation in CGD. Apoptotic cells (here shown for neutrophils, PMN) externalize phosphatidylserine (PS), which is recognized through PS-receptors on macrophages. This interaction enables the uptake of apoptotic cells by macrophages, a process termed “efferocytosis”. Successful efferocytosis leads to the secretion of the anti-inflammatory cytokine TGF-*β* by macrophages and thereby facilitates resolution of acute inflammation. Due to an impaired externalization of PS by CGD neutrophils and/or other neutrophil-macrophage interaction mechanisms, both apoptosis and efferocytosis have been described to be dysfunctional in CGD. This leads to unbalanced neutrophil necrosis with release of intracellular proteases/oxidants and persistence of (sterile) inflammation. Two pathways have recently been proposed to be involved in this impairment: PS/PSR interactions were found to trigger downstream pathways comprising IL-4 and PPAR*γ*, which are critically involved in the regulation of efferocytosis. Beyond that, impaired phagocytosis of apoptotic cells by CGD macrophages could be reversed by IFN-*γ* treatment, an effect that was mediated through NO production, endogenous TNF-*α* production, and Rac activation.

Apoptosis (programmed cell death) of inflammatory cells represents a physiological mechanism to prevent secondary uncontrolled necrosis and hyperinflammation followed by tissue damage. Apoptotic cells externalize phosphatidylserine (PS), which is recognized through PS-receptors. This interaction enables the uptake of apoptotic cells by phagocytes (for instance, macrophages), a process termed “efferocytosis” [12]. This controlled removal of apoptotic cells is of pivotal relevance for short-lived inflammatory cells, prototypically neutrophils. Efferocytosis leads to the secretion of the anti-inflammatory cytokine TGF-*β* by macrophages and thereby facilitates resolution of acute inflammation [13]. Both apoptosis and efferocytosis of apoptotic cells by macrophages have been reported to be impaired in CGD patients and/or mice (see Figure 1 for illustration). Constitutive apoptosis has been shown to be delayed in both human and murine CGD neutrophils due to impaired PS exposure. This may lead to unbalanced neutrophil necrosis with release of intracellular proteases/oxidants and an increased risk of developing lupus disease in CGD patients [14]. However, other groups could not determine any significant impairment of apoptosis in CGD monocytes [15] or neutrophils [16]. The latter group also observed higher rates of necrosis induced by specific bacteria in CGD neutrophils [16].

Besides apoptosis, also uptake/efferocytosis of apoptotic neutrophils has been shown to be impaired by murine CGD macrophages and has recently been shown to contribute to hyperinflammation in mice [17–19]. Two distinct mechanisms have been reported that play a role in the impaired removal of apoptotic cells by macrophages (Figure 1). Fernandez-Boyanapalli et al. demonstrated that the impaired phagocytosis of apoptotic cells by CGD macrophages could be reversed by IFN-*γ* treatment [18]. Further studies showed that the IFN-*γ* priming rescue effect was mediated through NO production, endogenous TNF-*α* production, and Rac activation.

Following up the consequences of the deficient PS exposure in CGD neutrophils, studies demonstrated that the impaired PS/PSR-dependent production of IL-4 resulted in reduced generation of 12/15-lipoxygenase and reduced activation of the transcription factor PPARgamma (peroxisome proliferator-activated receptor gamma) [17]. This leads to altered macrophage programming (M2 macrophage phenotype) and decreased efferocytosis in CGD macrophages. The authors showed that it was possible to overcome this IL-4-dependent defect in efferocytosis by injecting PS in CGD mouse models *in vivo*. However, the authors of these studies also discuss that the impaired clearance of apoptotic cells, observed in X-CGD, may not be contributable to the NADPH oxidase deficiency, but could be favoured by the cytokine microenvironment.

### 2.2. Innate Immune Receptors

Neutrophils are the key effector cells in antibacterial host defense and are in the focus of CGD research [20–23]. Effector functionality of neutrophils is orchestrated by innate immune receptors such as Toll-like receptors (TLRs) and complement receptors [21–23]. Neutrophils from CGD patients show lower expression levels of TLR5, TLR9, CD11b, CD18, CD35, and CXCR1 compared with those from healthy control subjects, whereas similar or increased receptor expressions are found in patients with bacterial pneumonia [24]. Reduced TLR5 expression resulted in impaired neutrophil activation by bacterial flagella, and reduced CD11b/CD18 expression is associated with impaired phagocytosis of *Staphylococcus aureus*.

TLR5 and CD18 expression levels correlate with disease severity in CGD patients. TLR5 and TLR9 expressions are higher in patients with residual NADPH oxidase activity. *In vitro *inhibition of the NADPH oxidase in control neutrophils decreases TLR5 and TLR9 expression and impairs TLR5 function. TLR5 expression correlates with the frequency of lymphadenitis in CGD patients, suggesting a clinically relevant role for TLR5 dysregulation in the course of CGD. Previous studies of CGD neutrophils have found a reduced expression of CD35 on neutrophils from patients with CGD compared to healthy controls and patients with recurrent infections [25]. When viewed in combination, these studies indicate that CGD neutrophils are not only restricted in terms of NADPH-mediated intracellular oxidative killing, but display distinct phenotypical and functional abnormalities. In particular, TLR5 and TLR9 are impaired in CGD through a mechanism linked to the deficient ROS production in these cells. Whether and how this dysbalance of innate immune receptors on CGD neutrophils contributes to inflammatory manifestations awaits further investigation.

### 2.3. ROS-Dependent Oxidization of T-Cell Membrane Proteins

Rats and mice with a lower capacity to produce ROS because of polymorphisms or mutations in the gene encoding the p47phox protein of the NADPH oxidase complex are more susceptible to develop severe arthritis [26, 27]. The lower capacity to produce ROS is associated with an increased number of reduced thiol groups on T-cell membrane surfaces. This influences activation and proliferation of T cells and the susceptibility to arthritis development [28]. In a recent study the Holmdahl group further showed that ROS-deficient macrophages can actually prime autoreactive T cells and initiate autoimmune arthritis in a murine model of collagen-induced arthritis [29].

### 2.4. Th17 Cells

**Figure 2:**
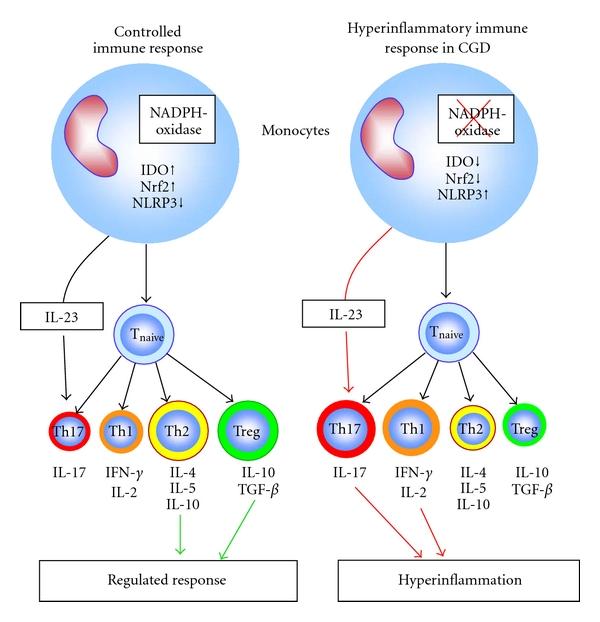
Other recently proposed mechanisms of hyperinflammation in CGD. Three independent mechanisms leading to hyperinflammation with increased numbers of Th17 cells have been recently proposed in CGD: Reduced IDO and Nrf2 activity and increased NLRP3 activation.

Recently, IL-17-producing effector cells (Th17 cells and *γδ* T cells) have been found to be involved in chronic inflammatory processes and several autoimmune diseases, for example, multiple sclerosis or rheumatoid arthritis. These highly proinflammatory cells are essential for the defence against pathogens [30, 31]. They are considered to be counterbalanced by regulatory T cells (Treg) [32]. A fine-tuned activity of both of these T-cell subsets is crucial for controlling infections, inflammation, autoimmunity, and malignancies [33, 34]. Evidence supporting a contribution of IL-17 in CGD hyperinflammation was recently derived from animal model data [35, 36]. When challenged with either intratracheal zymosan or LPS, NADPH oxidase-deficient p47phox^−/−^ mice and gp91phox^−/−^ mice develop exaggerated and progressive lung inflammation, augmented NF-kappaB activation, and elevated downstream proinflammatory cytokines (TNF-alpha, IL-17, and G-CSF) compared to wild-type mice [36]. Replacement of functional NADPH oxidase in bone marrow-derived cells restores the normal lung inflammatory response. Studies *in vivo* and in isolated macrophages have demonstrated that in the absence of functional NADPH oxidase, zymosan fails to activate Nrf2, a key redox-sensitive anti-inflammatory regulator. Consistent with these findings, zymosan-treated peripheral blood mononuclear cells from X-linked CGD patients show impaired Nrf2 activity and increased NF-kappaB activation [36] (Figure 2).

### 2.5. Indolamine-2,3-Dioxygenase

In addition to regulatory cells and their specific cytokines, immune responses are controlled by the enzyme indolamine-2,3-dioxygenase (IDO). IDO is required for the catabolism of the aromatic amino acid L-tryptophan and the generation of L-tryptophan metabolites. Whereas IDO-dependent metabolites of L-tryptophan like L-kynurenine are well-known immunosuppressive agents [37, 38], L-tryptophan itself or IDO-independent metabolites of tryptophan have been shown to activate proinflammatory Th17 cells [39]. IDO is primarily expressed in monocytes and dendritic cells [40]. The impact of IDO on immune regulation has become evident in the maternal tolerance to the allogeneic fetal tissue [41], in several autoimmune disorders like type-1 diabetes, multiple sclerosis, chronic inflammatory bowel disease, rheumatoid arthritis and systemic lupus erythematodes [37], as well as in the tolerance against malignant tumors [42]. Presently, a phase-III clinical trial with synthetic immunomodulatory tryptophan metabolites is being conducted in multiple sclerosis patients [43]. IDO function has been reported to be dependent on superoxides [35, 44], which are not produced in suitable amounts when the NADPH oxidase system is deficient as in CGD. IDO deficiency could thus be an important factor for the excessive inflammatory reactions in this disease (Figure 2). Recently, in p47phox^−/−^mice a pathogenic link between defective NADPH oxidase, reduced tryptophan catabolism, and IL-17-mediated inflammation [35] has been shown.After infection with *Aspergillus fumigatus*, these mice develop an enormous inflammatory response with a shift in the *γδ* T cell system and overproduction of IL-17 in comparison to wild-type mice. Furthermore, the hyperinflammatory phenotype of the CGD mice can be mimicked in wild-type mice by IDO blockade and abolished by replacement therapy with a natural kynurenine [35]. In humans, however, two independent studies recently showed, that monocyte-derived dendritic cells generated from patients harbouring X-linked and autosomal recessive forms of CGD, and from healthy controls, produced similar amounts of the tryptophan metabolite kynurenine upon activation with lipopolysaccharide and interferon-gamma [45, 46]. Thus, in humans, ROS apparently are dispensable for IDO activity and hyperinflammation in human CGD cannot be attributed to disabled IDO activation.

### 2.6. Inflammasome Activation

Some *in vitro* studies suggest that ROS are crucial for secretion of IL-1beta via inflammasome activation [47], whereas mice defective for ROS and patients with CGD have a proinflammatory phenotype. In three current studies, the activation of the IL-1beta inflammasome in cells from CGD patients was evaluated [48–50]. In contrast to previous studies using the small molecule diphenylene iodonium (DPI) as an ROS inhibitor, these studies did not find a decrease in either caspase-1 activation or secretion of IL-1beta and IL-18 in primary CGD monocytes. Moreover, activation of CGD monocytes by uric acid crystals induced a 4-fold higher level of IL-1beta secretion compared with that seen in control monocytes. This increase was not due to increased synthesis of the IL-1beta precursor. In addition, Western blot analysis of CGD cells revealed that caspase-1 activation was not decreased, but rather was increased compared with control cells [48] (Figure 2). Caspase-1 activation was especially strong in CGD patients with noninfectious inflammatory conditions. Treatment with IL-1 receptor antagonist reduced IL-1 production in monocytes ex vivo and during medical therapy [49]. These recent findings support the concept that ROS likely dampen inflammasome activation and identify phagocyte oxidase defective monocytes as a source of elevated IL-1. This provides new potential therapeutic options for inflammatory conditions associated with CGD.

## 3. Summary

CGD patients suffer from various autoinflammatory symptoms, such as granuloma formation, Crohn-like colitis, and lung fibrosis. As the majority of CGD patients reaches adulthood, autoinflammatory manifestations become more prominent and determine morbidity of many CGD patients. Here we summarize evidence on mechanisms underlying hyperinflammation in CGD. These include reduced neutrophil apoptosis, dysbalanced innate immune receptors, induction of Th17 cells, impaired Nrf2 activity, and increased inflammasome activation. Further studies are required to determine the clinical relevance of and, in particular, the therapeutic options for manipulation of these mechanisms in CGD patients.
